# Red Raspberry Seed Oil Low Energy Nanoemulsions: Influence of Surfactants, Antioxidants, and Temperature on Oxidative Stability

**DOI:** 10.3390/antiox11101898

**Published:** 2022-09-25

**Authors:** Ana Gledovic, Aleksandra Janosevic-Lezaic, Slobodanka Tamburic, Snezana Savic

**Affiliations:** 1 Department of Pharmaceutical Technology and Cosmetology, Faculty of Pharmacy, University of Belgrade, 11121 Belgrade, Serbia; 2 Department of Physical Chemistry and Instrumental Methods, Faculty of Pharmacy, University of Belgrade, 11121 Belgrade, Serbia; 3 Cosmetic Science Research Group, London College of Fashion, University of the Arts London, London WC1V 7EY, UK

**Keywords:** nanoemulsification, polyglycerol ester, polysorbate 80, natural, oregano oil, oak extract

## Abstract

The aim of this study was to assess and improve the oxidative stability of red raspberry seed oil–RO, a potential topical ingredient derived from food industry by-products, on its own and when incorporated in low energy nanoemulsion (NE). The RO’s oxidative stability was assessed at 5, 25, and 40 °C during one month of storage and expressed in: peroxide value, p-anisidine, and thiobarbituric reactive substances—TBARS value, while for NEs, lipid hydroperoxides and TBARS values were monitored. Both synthetic (butylated hydroxytoluene—BHT and ethylenediaminetetraacetic acid—EDTA), and natural (oregano essential oil—ORE and oak fruit extract—OAK) antioxidants were used. Pure RO and RO with BHT or ORE were stable at 5 °C and 25 °C, but at 40 °C BHT showed only moderate protection, while ORE was prooxidant. NEs prepared with new biodegradable polyglycerol esters-based surfactants, with droplet sizes of < 50 nm and narrow size distribution, showed improved physicochemical stability at room temperature, and especially at 40 °C, compared to NEs with polysorbate 80, which required the addition of antioxidants to preserve their stability. Natural antioxidants ORE and OAK were compatible with all NEs; therefore, their use is proposed as an effective alternative to synthetic antioxidants.

## 1. Introduction

Nowadays, growing popularity of natural products is evident in all health-related industries due to consumer preferences and ecological concerns, as well as marketing trends [1,2]. In particular, ingredients obtained from food industry waste during fruit and vegetable processing are being increasingly investigated in pharmaceutical and cosmetic industry, aiming to create safe products with added value [1,2,3]. One such by-product is red raspberry (*Rubus idaeus* L.) seed oil—RO, a “specialty oil” with potentially important dietary and functional properties. RO is recognized as a rich source of polyunsaturated fatty acids (PUFAs): C18:2 ω6 linoleic acid and C18:3 ω3 α-linolenic acid, with ω6: ω3 ratio from 1.5:1 to 2.7:1, which is considered a favorable ratio for the prevention of cardiovascular and carcinogenic diseases [4,5]. Moreover, there is a growing body of evidence that oral consumption of PUFAs, as well topical application of PUFA-loaded products, improve skin barrier function and vitality, leading to alleviation of various skin disorders accompanied with inflammation (i.e., photoaging, atopic dermatitis or psoriasis) [6,7].

Previous studies have shown that RO exhibits antioxidant activity [4,8,9,10], due to the presence of minor bioactive components such as carotenoids, tocopherols, and phenolic compounds [11,12]. However, despite these notable antioxidant properties, the shelf life of plant oils with high PUFA content remains limited (usually 6 to 12 months, if stored protected from light and air), due to their poor oxidative stability, especially at elevated temperatures [13,14]. Since natural antioxidants themselves exhibit instability, it would be useful to explore the use of innovative delivery systems as a strategy to ensure their stabilization, biocompatibility, and efficacy. In that regard, nanotechnological approach, especially nanoemulsions, has shown much promise recently [15,16,17]. 

In particular, nanoemulsions of the oil-in-water type (O/W NEs), constituted from oil phase dispersed in water phase in the form of small nanodroplets (usually from 20 to 200 nm) are suitable for encapsulation of lipophilic actives (such as RO). They provide various benefits (improved physical stability, prolonged or controlled release, pleasant visual appearance due to increased transparency) compared to other emulsion systems. Another benefit is a possibility to prepare NE systems by employing low energy methods, i.e., without high temperature or high-shear devices, which preserves quality of the natural and other sensitive ingredients [18,19]. The literature data regarding the lipid oxidation in classical emulsions are conflicting, since it was reported that lipid oxidation can be increased compared to bulk oil [20], but the opposite behavior was also described [21]. Due to the smaller size of nanodroplets compared to classical emulsions, the larger available oil–water interfacial surface area sometimes leads to even more pronounced oxidative instability [22,23], but the same controversy on the subject is evident in the reported results regarding NEs [24,25,26]. To avoid such problems, additional fine-tuning of the system composition may be required, based on elucidation of the role that surfactants and additional antioxidants play in the antioxidant stability of NE formulations [27,28]. 

Therefore, it was of interest to investigate the possibility of improving oxidative stability of RO in low energy O/W NEs prepared with novel polyglycerol ester-based mixture in comparison to polysorbate 80 as a standard small molecule surfactant in nanoformulations. This is a continuation of our previous work, where such systems were successfully developed and characterized, showing preliminary stability at room temperature and notable antioxidant properties [10,29,30]. Additionally, it was of interest to explore the feasibility of using natural antioxidants in NEs, specifically oregano seed oil (lipophilic) and oak extract (hydrophilic) instead of synthetic ones, since they are sometimes linked to adverse effects on human health [24,31,32]. Therefore, the aim of this study was to provide new insights into the antioxidant stability of nanoemulsions with natural ingredients, in order to contribute to the development of stable, safe, and efficient topical products. 

## 2. Materials and Methods

### 2.1. Materials

Nonionic surfactants: Polysorbate^®^ 80 (polyoxyethylene-20 sorbitan monooleate) produced by Fagron, Trikala, Greece and polyglycerol ester-based mixture prepared from Tego Care^®^ PL4 (polyglyceryl 4-laurate) and Tego Solve^®^ 61 (polyglyceryl-6 caprylate, polyglyceryl-3 cocoate, polyglyceryl-4 caprate, polyglyceryl-6 ricinoleate at 1:1:1:1 ratio). Mutual ratio of Tego Care^®^ PL4 to Tego Solve^®^ 61 was 6:4; these surfactants were produced by Evonik Nutrition and Care GmbH, Essen, Germany. Preservative mixture with additional cosurfactant properties was Euxyl PE^®^ 9010 (phenoxyethanol, ethylhexylglycerin), produced by Schülke GmbH, Norderstedt, Germany.

Oil phase components: Pelemol^®^ OPG (ethylhexyl pelargonate) was received as a free sample from Phoenix Chemical, Inc. Branchburg, NJ, USA; red raspberry (*Rubus idaeus*) seed oil—RO, cold pressed, unrefined oil contained 80% PUFAs out of 92% total unsaturated faffy acids (C18:2 ω6 linoleic —58.05%, C18:3 ω3 linolenic —22%, C18:1 ω9 oleic acid—12%, according to manufacturer specification) was produced by Aromaaz International, New Delhi, India. 

Water phase components: ultra-purified water was obtained with GenPure apparatus (TKA Wasseranfbereitungssysteme GmbH, Neiderelbert, Germany). The polyglycerol ester-based NEs also contained Glycerol Solvagreen^®^ (≥98%, anhydrous, Ph. Eur. grade), produced by Carl Roth GmbH, Karlsruhe, Germany; their pH value was adjusted with freshly prepared 0.1 M sodium hydroxide aqueous solution. 

Antioxidants: synthetic butylated hydroxytoluene (BHT) was produced by Sigma-Aldrich Co., St. Lois, MO, USA and ethylenediaminetetraacetic acid disodium salt dihydrate (EDTA) was produced by Lach-ner, Ltd., Neratovice, Czech Republic; both were obtained from local distributors. Two natural antioxidants were also used: Oregano (*Origanum vulgare*) essential oil—ORE containing 80% of carvacrol (produced by Aromaaz International, New Delhi, India, for domestic brand Eterra/company Terra Co, Novi Sad) and oak fruit (acorn) extract, Phytessence^®^ French oak—OAK (INCI: Aqua, Glycerin, Quercus petraea fruit extract), standardized at 1% polyphenols, received as free sample from Crodarom, Chanac, France. Lipophilic antioxidants (BHT or ORE) were added to the pure RO or to the NE oil phase, while the hydrophilic antioxidants (EDTA or OAK) were added to the NE water phase.

Reagents for determination of primary and secondary oxidation products in RO and nanaoemulsions: barium chloride dihydrate and ammonium thiocyanate were produced by Carl Roth GmbH, Karlsruhe, Germany; iron (II) sulfate heptahydrate, cumene hydroperoxide, thiobarbituric acid, trichloroacetic acid, butylated hydroxytoluene (BHT), 1,1,3,3-tetraethoxypropane (TEP), chloroform, 2-propanol, isooctane and 1-butanol were produced by Sigma-Aldrich Co., St. Lois, MO, USA. Hydrochloric acid was produced by Fischer Scientific, Loughborough, UK. Ethanol and methanol were produced by Honeywell, Muskegon, MI, USA. All solvents and reagents were of analytical grade or higher.

### 2.2. Methods

#### 2.2.1. Nanoemulsion Preparation

The NEs were prepared using the isothermal phase inversion composition (PIC) method suitable for heat-sensitive ingredients, at room temperature, without high-shear homogenization equipment or heating, which is recognized as low energy nanoemulsification due to considerable energy savings compared to the conventional production [18,19]. Briefly, the samples were prepared by stepwise addition of water phase with continuous vortex mixing (at 1300 rpm) to previously prepared surfactant-oil (SO) mixtures (2 min, at 1000 rpm). Formulation optimization of these RO-loaded NEs and the mechanism of nanoemulsification are described in detail in our previously published papers [10,29]. Therefore, for the purpose of the current study, similar formulations were prepared with polysorbate 80 (P80 NEs) or polyglycerol ester-based surfactants (PG NEs), with or without additional antioxidants (BHT—0.02 wt%, EDTA—0.2 wt%, ORE—0.02 wt% or OAK—1 wt%), while the nanoemulsion oil phase composition was kept constant. The antioxidants concentrations were chosen to be suitable for topical application, based on the common practice in cosmetic or pharmaceutical formulations, producer specifications, and the available literature [26,27,28,29,30,31,32]. After all NE components were added, a short homogenization of samples followed (for 2 min, at 1300 rpm) to obtain oil-in-water NEs containing 10 wt% surfactant, 10 wt% oil phase, and 80 wt% water phase, where wt% represents percentage weight by weight. The NE composition is presented in Table 1. The samples were kept in 10 mL glass bottles, capped tightly, and stored in the dark place for 30 days at different temperatures (5, 25, and 40 ± 2 °C, i.e., refrigeration, standard room temperature, and slightly elevated temperature, for example during summer, respectively), as a standard procedure to assess influence of the usual storage conditions for topical products and food [26,32].

#### 2.2.2. Nanoemulsion Characterization and Physical Stability

##### Particle Size Distribution 

The mean droplet size Z-average diameter (Z-ave) and droplet size distribution (polydispersity index—PDI) of the NEs were determined by dynamic light scattering (Zetasizer Nano ZS90, Malvern Instruments Ltd., Worcestershire, UK). Before measurement, each NE sample was diluted with ultra-pure water (1:100, *v*/*v*). The samples were measured in triplicate at 25 ± 2 °C, 24 h after production and after one month of storage at different temperatures (storage stability study).

##### Electrical Conductivity and pH Value Measurements 

Electrical conductivity of NEs was measured using SENSION+ EC71 apparatus (HACH, Loveland, CO, USA), while pH values were measured with HI2223 pH/ORP meter (Hanna Instruments Inc., Ann Arbor, MI, USA). The measurements were performed on undiluted NE samples, in triplicate, at 25 ± 2 °C according to the stability testing protocol.

### 2.3. Oxidative Stability Investigations

#### 2.3.1. Red Raspberry Seed Oil (RO) Oxidative Stability

For the purpose of oxidative stability investigations, lipophilic antioxidants were added to RO in the concentrations intended for skincare products—BHT at 0.2 wt% or 0.6 wt%, and ORE at 0.2 wt% or 1 wt%. The samples of pure RO or RO with added antioxidants (RO-BHT 0.2, RO-BHT 0.6, RO-ORE 0.2 and RO-ORE 1) were stored in tightly capped 5 mL glass bottles protected from light. The oil samples were analyzed initially (24 h after opening and/or addition of antioxidant), and again after 15 and 30 days of storage, at different temperatures (5, 25, or 40 ± 2 °C). The following parameters were analyzed: peroxide value (PV) as a measure of primary oxidation products, and p-anisidine value (PA) and thiobarbituric reactive substances (TBARS) value as measures of secondary oxidation products.

##### Peroxide Value (PV)

PV was determined according to the official International Dairy Federation method (IDF), a reliable spectrophotometric method suitable for small amounts of oil (≤0.01–0.3 g) which is convenient for fast analysis of numerous samples. The IDF method is based on the ability of lipid hydroperoxides to oxidize Fe(II) to Fe(III) ions, which form a deep red colored complex with thiocyanate anion [33]. The concentration of lipid peroxides can be quantified by measuring the absorbance of this complex at 500 nm after 5 min of reaction time; the calibration curve is usually prepared with Fe(III) solution. The preparation of reagents and analysis of oil samples was performed exactly as reported previously [33], and the calibration curve was prepared by analyzing standard samples containing 2.5–50 µg Fe(III) instead of oil samples. The results were expressed as milliequivalents of peroxide per kilogram of sample, calculated according to the following formula:

(1) $$\mathrm{PV}=\frac{\left(\mathrm{As}-\mathrm{Ab}\right)\times s}{55.84\times m\times 2}$$

where A_s_ is absorbance of the sample, A_b_ is absorbance of the blank (containing all reagents without the oil sample), s is slope, (in this experiment, s was 31.18), m is mass in grams of the oil sample, and 55.84 is atomic weight of iron. The division by factor 2 is necessary to express the peroxide value as milliequivalents of peroxide instead of milliequivalents of oxygen, as mentioned in reference [33]. The absorbances were measured via the Evolution 300 UV-VIS spectrophotometer (Thermo Scientific, Loughborough, UK).

##### p-Anisidine Value (PA)

The method used to determine the PA of oil samples was based on the procedure previously described [32] and according to the AOCS official method Cd 18-90 [34] with slight modifications regarding the reduction of amount of oil and reagents. The reaction is based on the reaction between p-anisidine and aldehydic compounds (mainly 2-alkenals and 2,4-alkadienals) in oils and fats in acidic conditions, resulting in the formation of a yellow-colored compound with absorbance at 350 nm. Briefly, p-anisidine reagent was prepared by dissolving of p-anisidine (0.025 g) in 10 mL of glacial acetic acid. Next, 0.5 g of oil sample was dissolved in isooctane and filled to 25 mL in a volumetric flask. First, the unreacted solution was prepared by mixing 2.5 mL of the oil solution and 0.5 mL of glacial acetic acid in a glass vial (for 10 s, with vortex mixer). The other 2.5 mL of the oil solution was mixed with 0.5 mL of p-anisidine reagent in another glass vial and kept in the dark for 10 min (reacted solution). The absorbance of these solutions and the blank solution (isooctane 2.5 mL mixed with 0.5 mL of p-anisidine reagent) were measured at 350 nm against isooctane, using a UV-VIS spectrophotometer. For the calculation of PA, the following equation was used:

(2) PA=25×[1.2×(A2−Ab)−A1]÷m

where A1 is the absorbance of unreacted solution, A2 is the absorbance of reacted solution, and Ab is the absorbance of the blank, m is the mass of oil sample. The measurements were performed in triplicate.

##### Thiobarbituric Reactive Substances (TBARS)

TBARS measurements were carried out following the previously described procedure [35] with slight modifications. In brief, the oil samples (0.1 g, i.e., 120 µL) were mixed (for 10 s, with vortex mixer) with 3.75 mL of the freshly prepared TBA reagent containing 15% *w*/*v* trichloroacetic acid and 0.375% *w*/*v* thiobarbituric acid, dissolved in 0.25 M HCl and 600 µL 2% *w*/*v* BHT solution in ethanol, and filled up with ultra-purified water up to 5 mL in screw-capped glass test tubes. The reaction mixtures were then heated in the water bath at 90 ± 5 °C for 15 min, followed by quick cooling in tap water to room temperature and centrifuged for 15 min at 3000 rpm (MPW centrifuge, MPW MED. Instruments, Warszawa, Poland). After 10 min, the absorbance of the supernatant containing pink colored complex formed from TBA and malonaldehyde was measured at 532 nm. The blank sample was prepared with ultra-pure water instead of oil, and its absorbance was subtracted from the measured absorbances of the samples. The TBARS content (expressed as mg malonaldehyde per kilogram of oil (MDA/kg oil) was obtained from the calibration curve prepared with 1,1,3,3-tetraethoxypropane (TEP) as a standard compound. 

#### 2.3.2. Nanoemulsion Oxidative Stability

##### Determination of Lipid Hydroperoxides (LH) in Nanoemulsions

The formation of the primary oxidation products in the NE samples (blank NE and RO-loaded NEs with or without additional antioxidants), as well as in the surfactant dispersion in water phase without oil was performed according to the method described by Walker et al. [35] with slight modifications. First, the Fe(II) solution was freshly prepared prior to analysis, by mixing equal amounts of 0.132 M barium chloride and 0.144 M ferrous sulfate solutions in ultra-pure water, followed by centrifugation at 5000 rpm for 5 min to obtain a clear solution. The lipid extraction from NE samples was performed by mixing 0.2 mL of NE with 1 mL of isooctane/isopropanol (3:1 *v*/*v*), followed by vortexing for 10 s, and then centrifugation at 3000 rpm, for 2 min. The top layer (0.2 mL) was then collected and mixed with 2.8 mL of methanol/1-butanol (2:1 *v*/*v*), followed by addition of 15 µL of Fe(II) solution and 15 µL of ammonium thiocyanate solution as activator (3.94 M). The reaction mixtures were kept protected from light, and the absorbance was measured after 20 min against methanol/butanol mixture. The blank sample was prepared with isooctane instead of NE extract, and its absorbance was subtracted from the absorbance of the samples. For the method validation, cumene hydroperoxide was used as standard compound, and the results were expressed as mmol cumene hydroperoxide/L nanoemulsion (mmol CUM/L nanoemulsion).

##### Determination of TBARS in Nanoemulsions

TBARS values of NEs were determined when 500 µL of NE was mixed with 4 mL of the TBA reagent and 500 µL of BHT solution, by following the same procedure as described for the oil samples. The results were expressed as mg MDA/L nanoemulsion.

### 2.4. Statistical Analysis

Results are presented as means and standard deviations (SD) of measurements conducted in triplicate. The influence of added antioxidants on the droplet sizes and PDI values of NEs was investigated by one-way analysis of variance (ANOVA) using OriginPro8.5 (Originlab Corporation, Northampton, MA, USA). Evaluation of the effect of two independent variables (i.e., different antioxidants and different storage temperatures) on the oxidative stability of oils and NEs was performed via the two-way analysis of variance for different time intervals (15 and 30 days), followed by Tukey’s post hoc test, while *p*-value of <0.05 was considered statistically significant.

## 3. Results and Discussion

### 3.1. Influence of Red Raspberry Seed Oil, Antioxidants, and Temperature on Nanoemulsion Properties

#### 3.1.1. The Nanoemulsion Droplet Size Distribution 

Nanocarriers, in particular O/W NEs produced via low-energy methods are being increasingly proposed as promising carriers for natural and sensitive lipophilic ingredients such as natural oils and extracts, aiming to preserve/improve their stability and efficacy [19,36,37]. As previously described in the literature, the low-energy nanoemulsification (such as the PIC method used in this study) is based on the release of chemical energy when selected ingredients are mixed in a specific way, leading to the formation of small nanodroplets due to surfactant self-assembly [18,19,38]. Therefore, every change in system composition, such as the choice of surfactants, or addition of natural oil or other lipophilic or hydrophilic additives (i.e., antioxidants) can significantly influence NE properties and their stability [18,36]. 

For the purpose of this study, two different non-ionic surfactants: polysorbate 80—a standard surfactant suitable for various applications [23,39] and polyglycerol ester-based mixture—a novel biocompatible and biodegradable surfactant blend also suitable for food and cosmetics [40,41], were chosen to prepare NEs (Table 1). In our previous studies, NEs with similar composition were thoroughly optimized and characterized [10,29]. However, to the best of our knowledge, the influence of polyglycerol-ester based surfactants on NE physicochemical stability with additional antioxidants at different storage temperatures has not been reported before, neither it was compared to polysorbate 80 as the traditionally used stabilizer in submicron/NE formulations.

As it can be seen from Table 2, the choice of surfactant and addition of RO both had a significant impact on NE properties, while the addition of lipophilic (BHT or ORE) or hydrophilic antioxidants (EDTA or OAK) did not have significant effect. Namely, 24 h after preparation, RO-loaded P80 NEs had droplet sizes ranging from 203.50 nm to 211.43 nm, while F0 P80 without RO and antioxidants had bigger droplets (245.3 nm). As a result, all P80 NEs were milky white with slight bluish tone, indicative of NEs [42]. On the other hand, PG NEs were semi-transparent, due to very fine droplet sizes (Z-ave < 65 nm), also with notable differences between blank NE (F0 PG: 62.32 nm) and RO-loaded NEs (38.63–46.56 nm). Droplet size distribution was very narrow (PDI < 0.15) in both types of NE systems (i.e., 0.116–0.125 for P80 NEs, and 0.051–0.110 for PG NEs, respectively), indicating good preliminary stability [26,43]. 

**Figure 1:**
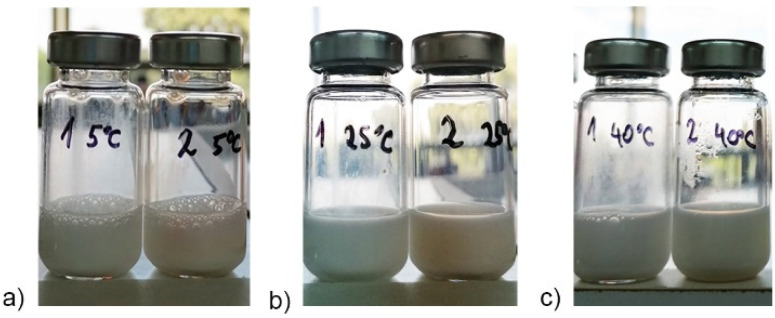
Visual appearance of nanoemulsions prepared with polysorbate 80 without raspberry oil on the left (sample 1—F0 P80 NE, where creaming process is visible on the glass vial wall) and with raspberry oil on the right (samples 2—F1 P80 NE, with improved stability) stored at different temperatures: (**a**) 5 °C, (**b**) 25 °C, and (**c**) 40 °C.

After 30 days of storage at different temperatures, both systems showed satisfactory preliminary stability according to droplet size measurement, since there was no significant increase in droplet sizes for the majority of samples and PDI values remained without significant changes (Table S1, Supplementary file). However, visual observations (Figure 1) revealed that some changes occurred in P80 NEs that could not be detected via the droplet size measuring instrument due to its limited range (up to 5 microns) and because of the dilution prior to measurements. Although a standard procedure, the dilution could prevent the detection of some common instability phenomena (i.e., flocculation and formation of larger aggregates) [42]. For example, the creaming process obviously occurred in blank P80 formulation at all temperatures, indicating that addition of RO had a stabilizing effect on P80 NEs (Figure 1). 

**Figure 2:**
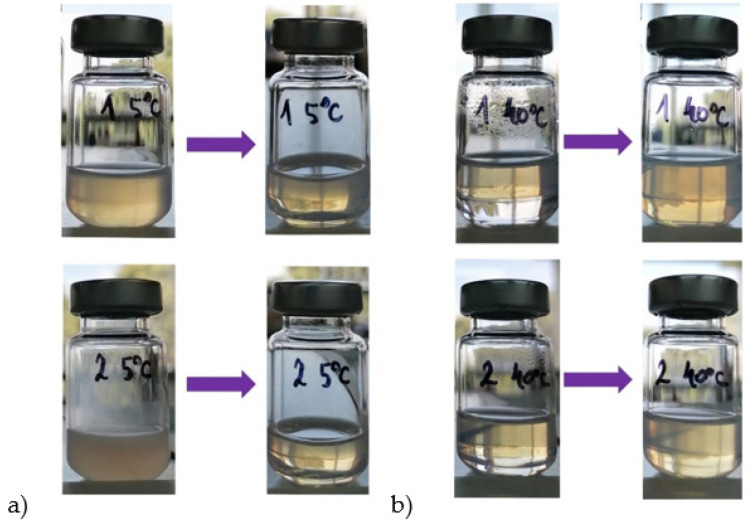
Visual appearance of nanoemulsions prepared with polyglycerol ester-based mixture without red raspberry oil on the left (sample 1—F0 PG NE) or with red raspberry oil on the right (sample 2—F1 PG NE) after one month of storage: (**a**) at 5 °C (cloudy samples), (**b**) at 40 °C (2-phase samples). All samples became clear/semi-transparent after one hour at room temperature (RT).

In the case of PG NEs, some interesting properties were revealed: for example, at 5 °C most of PG NEs became cloudy, while at 40 °C they became 2-phase systems (most likely a concentrated NE on the top with Z-ave 52.23 nm, PDI 0.18, and with an excess water phase below). However, these transformations of PG NEs were reversible, since after a few hours at RT and slight shaking (for 1 min) all PG NEs spontaneously recovered to their original state, with very similar droplet size distribution as initially (Figure 2a,b, Table S1, Supplementary file). 

#### 3.1.2. The pH Value and Electrical Conductivity 

Further differences in stability among the NEs stabilized with different surfactants were manifested as changes in their pH value and electrical conductivity (data shown in Table S2, Supplementary file). It was observed that RO-loaded PG NEs exhibited good stability at all tested temperatures, since there were no major changes in their pH values, having in mind that decline in pH value is an indicator of fatty acid leakage from the nanoemulsion core [23,44]. Some changes in electrical conductivity were observed in most samples: up to 20% (for F1 PG+EDTA and F1 PG+OAK), up to 55% (for samples F1 PG+BHT, F1 PG+ORE), and especially for blank PG NE at all temperatures (up to 71% increase), although this was not reflected in overall stability of the samples. On a contrary, P80 NEs exhibited high instability at 40 °C, with very pronounced decrease of pH values (from 5.37 to 3.39 and from 5.45 to 3.72 for blank F0 P80 NE and F1 P80 NE with RO without added antioxidants, respectively). This can be attributed to low stability of P80 and natural oils with high PUFA content at elevated temperatures, as previously reported [23]. However, all tested antioxidants successfully prevented this NE deterioration, proving that it is necessary to add antioxidant to this type of surfactant. 

### 3.2. Oxidative Stability of Red Raspberry Seed Oil 

RO used in this study was an organic, cold-pressed, unrefined seed oil, known for its high PUFA content (>80%) and minor bioactive compounds (i.e., carotenoids and tocopherols), with orange color and pleasant herbal scent. In our previous investigations, this particular RO exhibited antioxidant activity per se or in different NE formulations [10,29]. However, similar to other natural oils with high PUFA content, there are concerns regarding the oxidative stability of RO during storage, especially at elevated temperatures, due to the fast formation of oxidation products, with potential hazardous effects on human health. Additionally, deterioration of natural oils leads to poor sensory attributes of products, such as rancid odor, change in color and texture [23,31,45]. Therefore, the first step was to investigate the oxidative quality of pure RO as a raw material, but also with added standard synthetic antioxidant (BHT) or ORE as a natural essential oil with remarkable antioxidant activity [30,46,47]. 

**Figure 3:**
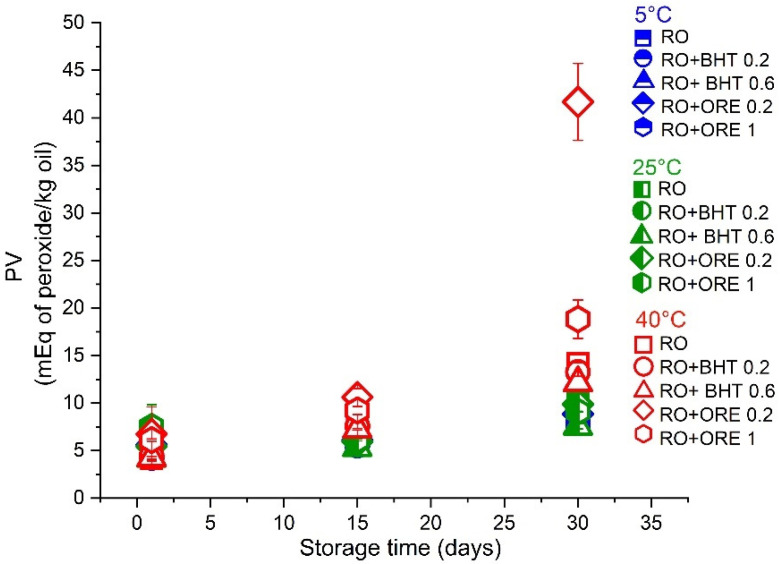
Peroxide values of oil samples (pure raspberry oil–RO) and RO with added antioxidants (butylated hydroxytoluene—BHT, 0.2 or 0.6 wt%, and oregano essential oil—ORE, 0.2 or 1 wt%) at 5, 25, and 40 °C during one month of storage. The results are expressed as milliequivalents of peroxide/kg oil, determined according to the IDF method.

It is well established that the lower the PV the better is the oil quality, while the PV value above 10 is an indicator of oil oxidation (formation of primary oxidation products, such as lipid hydroperoxides) [31]. In the case of cold-pressed oils, this limit goes up to 15 [13]. It can be seen from Figure 3 that pure RO and RO with all tested antioxidants exhibited satisfactory PV values below 10 during the first 15 days of storage at all tested temperatures. However, at the end of storage stability study, at 40 °C the majority of samples exceeded the limit of PV = 10, but still remained at a relatively satisfactory level (<20). Interestingly, it was also observed that ORE (at 0.2 wt%) acted as prooxidant, with a very prominent rise of PV (to 41.34). In conclusion, there were no significant differences among PV values of pure oil, and RO with added BHT (0.2 and 0.6 wt%), as well as among samples stored at 5 and 25 °C, which remained in the desired PV limits.

**Figure 4:**
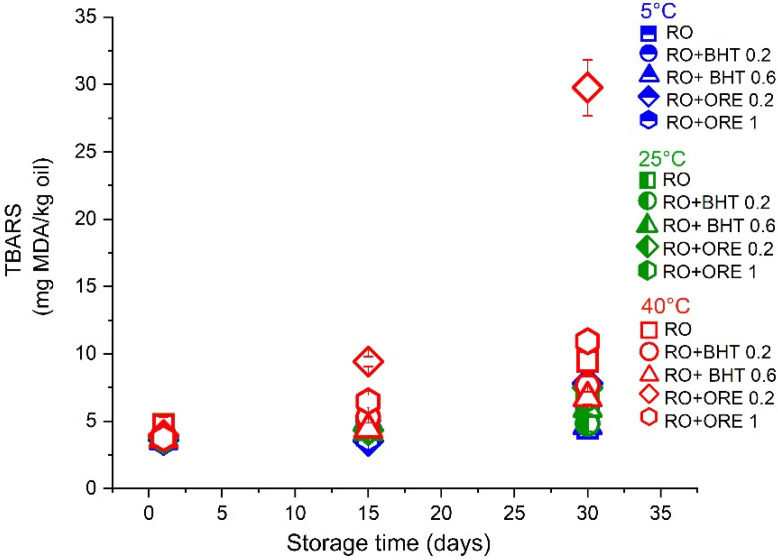
TBARS values of oil samples (pure raspberry oil–RO) and RO with added antioxidants (butylated hydroxytoluene—BHT, 0.2 or 0.6 wt%, and oregano essential oil—ORE, 0.2 or 1 wt%) at 5, 25, and 40 °C during one month of storage. The results are expressed as mg of malonaldehyde/kg oil (mg MDA/kg oil).

**Figure 5:**
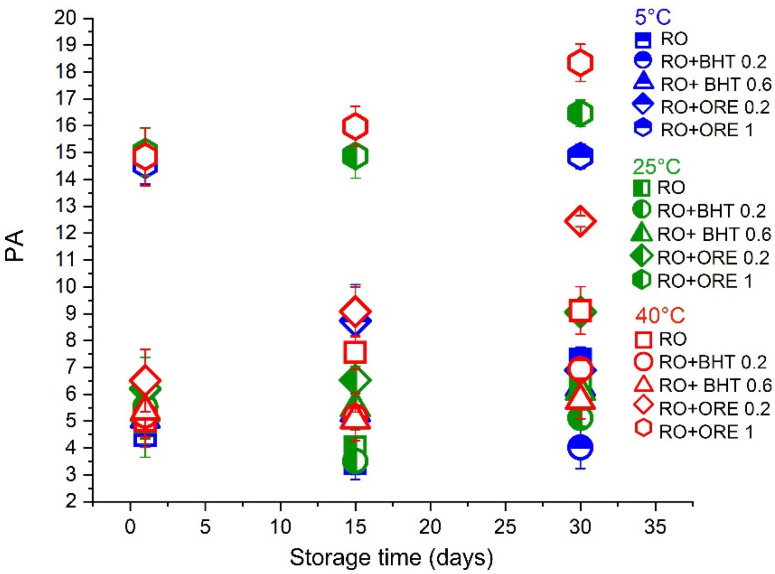
p-anisidine value (PA) of oil samples (pure raspberry oil–RO) and RO with added antioxidants (butylated hydroxytoluene—BHT, 0.2 or 0.6 wt%, and oregano essential oil—ORE, 0.2 or 1 wt%) during one month of storage at 5, 25, and 40 °C.

The formation of secondary oxidation products, such as malonaldehyde, was monitored via the TBARS test (Figure 4) and the formation of other aldehydes (i.e., 2-alkenals and 2,4-alkadienals) was expressed as PA (Figure 5).

Similar to the results of PV measurements, TBARS values at day 15 at all tested temperatures remained ≤5 mg MDA/kg oil for pure RO and RO-BHT 0.2 and 0.6 wt%, indicating slow degradation of the formed lipid hydroperoxides. However, there was a significant increase of TBARS in RO-ORE samples stored at 40 °C—from 3.96 to 9.40 (for 0.2 wt% ORE) and from 3.74 to 6.47 (for 1 wt% ORE), which was in line with the rise of PV in these samples. Finally, at 30 days of storage, there were no significant differences among samples stored at 5 and 25 °C, neither among samples of pure RO and RO with BHT (0.2 and 0.6 wt%) which had moderately increased TBARS. However, RO-ORE samples at both tested concentrations exhibited notable increase of TBARS.

The results of PA measurements (Figure 5) were also generally in line with the PV and TBARS values, since PA values of RO with or without added BHT remained below the recommended limit of 10 [13] at all tested temperatures after 30 days of storage, and there were no significant differences among samples stored at 5 and 25 °C. Interestingly, RO with 1 wt% ORE showed high PA even on the first day of the study, indicating the possibility that some minor component of this essential oil interacted with the test results. 

To conclude, it was found that oxidative stability of pure RO and RO with added antioxidants was satisfactory during one month of storage at 5 and 25 °C, with all investigated parameters (PV, PA, and TBARS) within optimal limits, which can be attributed to antioxidant activity of RO per se [10]. However, at 40 °C the BHT sample (at both tested concentrations) showed only moderate protection against oxidation, while RO-ORE samples showed high instability.

### 3.3. Oxidative Stability of Nanoemulsions with Red Raspeberry Seed Oil

As was previously reported, incorporation of oils into classical emulsions (with oil droplets in the micrometer range) and nanoemulsions (preferably up to ~200 nm) can have a controversial outcome on the oil oxidative stability. One of the main reasons for increased instability of oil in emulsion system compared to bulk oil is a large contact area between the oil and the water phase containing prooxidants. Moreover, previous studies showed that structural and physicochemical properties of surfactants, as well as their interactions with antioxidants in formulations, significantly influence oxidative stability of emulsified oils [23,25,26,45,48].

**Figure 6:**
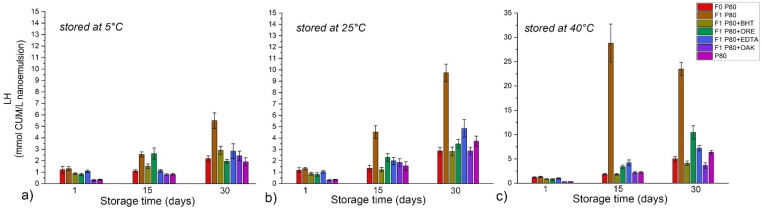
Lipid hydroperoxides—LH of polysorbate 80 dispersion in water (P80) and P80-stabilized NEs during one month of storage at different temperatures: (**a**) 5 °C, (**b**) 25 °C and (**c**) 40 °C. Blank nanoemulsion was prepared without raspberry seed oil—RO (F0 P80), while RO loaded NEs are marked as F1 P80; antioxidants tested in nanoemulsions were marked as: butylated hydroxytoluene—BHT, oregano essential oil—ORE, ethylenediaminetetraacetic acid disodium salt dihydrate—EDTA, or sessile oak extract—OAK.

**Figure 7:**
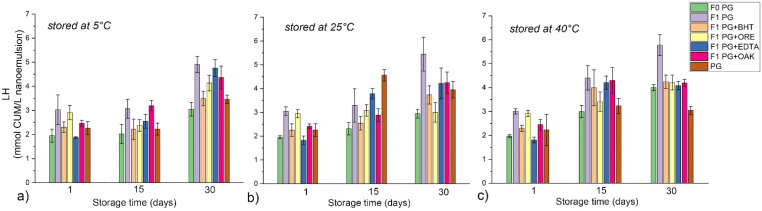
Lipid hydroperoxides—LH of polyglycerol ester-based mixture in water (PG) and PG-stabilized NEs during one month of storage at different temperatures: (**a**) 5 °C, (**b**) 25 °C, and (**c**) 40 °C. Blank nanoemulsion was prepared without raspberry seed oil—RO (F0 PG), while RO-loaded NEs are marked as F1 PG; antioxidants tested in nanoemulsions were marked as: butylated hydroxytoluene—BHT, oregano essential oil—ORE, ethylenediaminetetraacetic acid disodium salt dihydrate—EDTA, or sessile oak extract—OAK.

**Figure 8:**
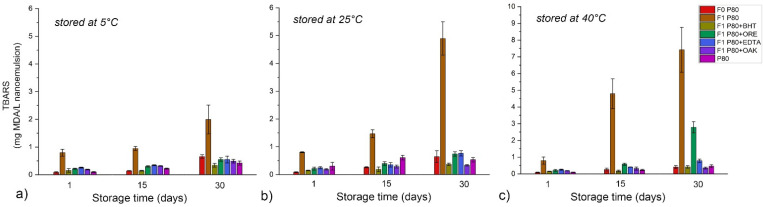
Thiobarbituric reactive substances—TBARS of polysorbate 80 dispersion in water (P80) and P80-stabilized NEs during one month of storage at different temperatures: (**a**) 5 °C, (**b**) 25 °C, and (**c**) 40 °C.

**Figure 9:**
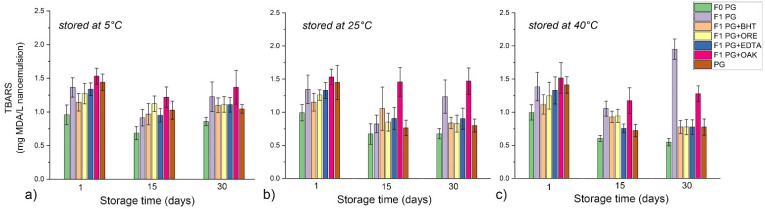
Thiobarbituric reactive substances—TBARS of polyglycerol ester-based mixture in water (PG) and PG-stabilized NEs during one month of storage at different temperatures: (**a**) 5 °C, (**b**) 25 °C, and (**c**) 40 °C.

The results of the formation of lipid hydroperoxides—LH as primary oxidation products for the NEs stabilized with polysorbate 80—P80 and polyglycerol ester-based mixture—PG are shown in Figure 6 and Figure 7, respectively. The formation of secondary oxidation products expressed as TBARS for the same two sets of samples are shown in Figure 8 and Figure 9. The results indicate that surfactants played a decisive role in nanoemulsion oxidative stability and that the addition of antioxidants may be necessary to optimize it at different storage temperatures.

P80 NE prepared without additional antioxidants (F1 P80) showed very high oxidative instability when stored at 40 °C, with a dramatic increase of LH and TBARS after only 15 days of storage (from 1.32 to 28.81 and from 0.79 to 4.79, for LH and TBARS, respectively) resulting in a distinctive rancid odor. This high instability can be attributed to synergistic interaction between RO and F1 P80 components, because the dispersion of pure surfactant (P80) and blank NE without RO both exhibited much slower formation of oxidation products. Such behavior was previously reported [49], and it was concluded that hydrophilic head groups in P80 molecules can generate hydroperoxides when in contact with prooxidants in water, resulting in faster oxidation of unsaturated fatty acids in oils. In this study it was shown that the addition of any of the tested antioxidants (especially BHT, OAK or EDTA) or storage in refrigerator significantly inhibited the nanoemulsion oxidation. 

Although in PG NEs LH values slightly increased with storage time, statistical analysis has revealed that after 30 days of storage there were no significant differences in LH values among the samples stored at three tested temperatures, and no significant differences in TBARS values among the samples stored at 25 °C and at 40 °C. Even in the case of the formulation F1 PG without additional antioxidants, LH and TBARS remained within acceptable limits, proving that the surfactant layer played an important role in the protection of RO within the NE core. As it was shown in Figure 2, at 40 °C PG-NEs transformed into a two-phase system, where RO was located in the top layer (yellowish colored concentrated nanoemulsion), while the remaining water phase was in the bottom. Therefore, this transformation could have played a role in RO stabilization, for example, by separating RO from prooxidants in the NE water phase or by thicker surfactant layer, although additional experiments should be performed to prove this hypothesis. As for the tested antioxidants, BHT, EDTA, and ORE showed similar positive effect on the oxidative stability of PG NEs, while OAK was the least effective. ORE could be used as antioxidant in both tested types of nanoemulsions, although it clearly exhibited prooxidative activity in combination with bulk RO. Therefore, its antioxidant activity was successfully preserved in a nanoemulsion carrier, which is in line with our previous findings where it was revealed that antioxidant activity of essential oils can be significantly changed when incorporated into nanoemulsion carriers [30].

It should be noted that PG NEs had four-times lower main droplet size diameter compared to P80 (~45 nm compared to ~210 nm) but the increase in total oil-to-water surface area did not decrease NE stability. Other researchers also concluded that droplet sizes cannot be taken as a single factor in NE oxidative stability, due to the complexity of nanoemulsion composition and possible interactions among ingredients such as oil phase components and antioxidants [25,26,50]. PG esters are prepared by esterification of natural glycerol with fatty acid from natural oils with different chain length and unsaturation level [40,41]. In our study, a commercially available mixture comprised of polyglyceryl 4-laurate, polyglyceryl-6 caprylate, polyglyceryl-3 cocoate, polyglyceryl-4 caprate, polyglyceryl-6 ricinoleate. Therefore, it can be expected that this complex composition contributed to the more compact surfactant packing and improved barrier at the oil–water interface compared to P80 (polyoxyethylene-20 sorbitan monooleate) as a single surfactant with small molecular weight [51]. This PG surfactant layer probably slightly limited the action of antioxidants at the oil–water interface; therefore, the addition of the tested antioxidants was less pronounced than the protective role of surfactant itself [52]. It is worth mentioning that the majority of papers presented oxidative stability of NEs calculated as mmol or mg CUM and MDA/kg of oil [25,26,28,50]. Having in mind that nanoemulsions have complex composition, with multiple possible interactions among ingredients, we have expressed the results as mmol CUM/L and mg MDA/L of nanoemulsions, as reported previously [35,53]. However, as pointed out in publication involving fish oil in conventional vs. nanoemulsions NEs [54], the limits for LH and TBARS values for (nano)emulsions are yet to be determined. To the best of our knowledge, this study reports the oxidative stability of PG NEs for the first time. The results obtained are promising and could be applied to the products in the cosmetic, pharmaceutical, and food industries.

## 4. Conclusions

In this comprehensive study, we have investigated the simultaneous effect of the compositional factors (type of surfactant, oil phase composition, and addition of antioxidants) and temperature (5, 25, or 40 °C) on the physicochemical stability of low energy nanoemulsions, aiming to improve oxidative stability of red raspberry seed oil with high PUFA content. Pure red raspberry seed oil exhibited satisfactory oxidative stability during the first 15 days at all tested temperatures, which can be attributed to its innate antioxidant properties, while slight deterioration was evidenced after 30 days of storage at 40 °C. RO was compatible with BHT, which showed moderate protection against lipid oxidation, whereas ORE acted as prooxidant. Our results have shown that the nanoemulsification of RO with traditional polyethoxylated surfactant polysorbate 80 enhances the oxidative degradation of the oil, necessitating the use of antioxidants. This is in contrast with the effect of polyglycerol ester mixture, which stabilizes RO. It was found that polyglycerol ester-based surfactants, a new generation of biodegradable and biocompatible surfactants suitable for applications in food, medicine, and cosmetics, could be used to obtain nanoemulsions with very fine droplet sizes of < 50 nm and narrow droplet size distribution of PDI < 0.11. Moreover, PG NEs showed improved physicochemical stability at room temperature, and especially at 40 °C, compared to the P80 NE. It was also shown that natural antioxidants ORE and OAK are compatible with nanoemulsion carriers used in this study. Therefore, their use is proposed as an effective natural alternative to the synthetic molecules such as BHT and EDTA in natural formulations. 

## Figures and Tables

**Table 1:** Composition of nanoemulsions prepared with different surfactants.

Formulation Name	Surfactant (10 wt%)	Oil Phase (10 wt%)	Water Phase (80 wt%)
F0 P80		9.5 EP, 0.5 EUX	Water
F1 P80		7.5 EP, 2 RO, 0.5 EUX	Water
F1 P80 + BHT	P80	7.48 EP, 2 RO, 0.5 EUX, 0.02 BHT	Water
F1 P80 + ORE		7.48 EP, 2 RO, 0.5 EUX, 0.02 ORE	Water
F1 P80 + EDTA		7.5 EP, 2 RO, 0.5 EUX	Water—79.8, EDTA—0.2
F1 P80 + OAK		7.5 EP, 2 RO, 0.5 EUX	Water—79.0, OAK—1.0
F0 + PG		9.5 EP, 0.5 EUX	Water—76, Glycerol—4
F1 + PG		7.5 EP, 2 RO, 0.5 EUX	Water—56, Glycerol—24
F1 PG + BHT	PG	7.48 EP, 2 RO, 0.5 EUX, 0.02 BHT	Water—56, Glycerol—24
F1 PG + ORE		7.48 EP, 2 RO, 0.5 EUX, 0.02 ORE	Water—56, Glycerol—24
F1 PG + EDTA		7.5 EP, 2 RO, 0.5 EUX	Water—55.8, Glycerol—24, EDTA—0.2
F1 PG + OAK		7.5 EP, 2 RO, 0.5 EUX	Water—55.8, Glycerol—24, OAK—1.0

Table abbreviations: BHT—butylated hydroxytoluene, EDTA—ethylenediaminetetraacetic acid, disodium salt dihydrate, EP—ethylhexyl pelargonate, EUX—preservative/ cosurfactant mixture: phenoxyethanol, ethylhexylglycerin, OAK—oak fruit (acorn) extract, ORE—oregano essential oil, PG—mixture of polyglycerol ester-based surfactants: Tego Care^®^ PL4 and Tego Solve^®^ 61 at 6:4 ratio, P80—polysorbate 80, RO—red raspberry seed oil.

**Table 2:** Z-average droplet size (Z-ave), polydispersity index (PDI), pH value, and electrical conductivity (El. cond.) of nanoemulsions stabilized with different surfactants and antioxidants.

Formulation Name	Z-ave (nm)	PDI	pH	El. Cond. (µS/cm)
F0 P80	245.30 ± 3.12	0.121 ± 0.015	5.37 ± 0.006	159.33 ± 1.71
F1 P80	207.63 ± 1.46	0.108 ± 0.017	5.45 ± 0.015	156.03 ± 0.76
F1 P80 + BHT	210.03 ± 1.62	0.125 ± 0.004	5.38 ± 0.021	167.37 ± 0.65
F1 P80 + ORE	203.50 ± 1.15	0.108 ± 0.014	5.40 ± 0.015	155.07 ± 0.49
F1 P80 + EDTA	206.03 ± 1.39	0.084 ± 0.027	4.98 ± 0.058	744.67 ± 0.58
F1 P80 + OAK	211.43 ± 1.66	0.116 ± 0.020	6.16 ± 0.025	242.67 ± 2.08
F0 PG	62.32 ± 0.32	0.060 ± 0.014	4.16 ± 0.006	167.73 ± 0.40
F1 PG	41.96 ± 0.72	0.059 ± 0.018	4.19 ± 0.010	81.07 ± 1.51
F1 PG + BHT	38.36 ± 0.73	0.110 ± 0.008	4.20 ± 0.017	75.77 ± 0.68
F1 PG + ORE	41.40 ± 0.95	0.079 ± 0.016	4.21 ± 0.026	71.27 ± 0.75
F1 PG + EDTA	46.56 ± 1.01	0.051 ± 0.013	4.59 ± 0.060	346.67 ± 1.53
F1 PG + OAK	41.57 ± 0.83	0.069 ± 0.024	4.35 ± 0.056	89.85 ± 0.23

## Data Availability

Data is contained within the article and supplementary material.

## Supplementary Materials

The following supporting information can be downloaded at: https://www.mdpi.com/article/10.3390/antiox11101898/s1. Table S1. Z-average droplet sizes (Z-ave) and PDI values polysorbate 80 (P80) and polyglycerol ester-based (PG)-based nanoemulsions during one month of storage at different temperatures. Table S2. pH values and electrical conductivity of P80 and PG-based nanoemulsions during one month of storage at different temperatures.
