# Supplementary material for: Red Raspberry Seed Oil Low Energy Nanoemulsions: Influence of Surfactants, Antioxidants, and Temperature on Oxidative Stability
Source: Antioxidants (Basel). 2022 Sep 25;11(10):1898. doi: 10.3390/antiox11101898 (PMC9598911; doi:10.3390/antiox11101898)
Supplement: Supplementary file 1 [file antioxidants-11-01898-s001.zip › antioxidants-1923946-supplementary.pdf]

Supplementary file

Red Raspberry Seed Oil Low Energy Nanoemulsions: Influence of Surfactants, Antioxidants and Temperature on Oxidative Stability

Table S1. Z-average droplet sizes (Z-ave) and PDI values of polysorbate 80 (P80) and polyglycerol ester-based (PG)-based nanoemulsions during one month of storage at different temperatures.

	Z-ave (nm)			PDI		
**Formulation name**	5 °C	25 °C	40 °C	5 °C	25 °C	40 °C
**F0 P80**	211.73 ± 3.617	283.03 ± 5.532	230.77 ± 0.404	0.147 ± 0.020	0.206 ± 0.034	0.085 ± 0.023
**F1 P80**	219.33 ± 4.332	208.67 ± 1.501	207.33 ± 1.201	0.180 ± 0.021	0.078 ± 0.010	0.097 ± 0.015
**F1 P80 + BHT**	205.03 ± 2.354	207.80 ± 0.529	202.07 ± 1.501	0.106 ± 0.018	0.097 ± 0.037	0.097 ± 0.018
**F1 P80 + ORE**	199.87 ± 0.710	201.33 ± 1.358	198.80 ± 0.436	0.103 ± 0.019	0.091 ± 0.020	0.098 ± 0.045
**F1 P80 + EDTA**	203.90 ± 2.751	202.90 ± 2.778	201.47 ± 1.097	0.070 ± 0.018	0.068 ± 0.012	0.083 ± 0.027
**F1 P80 + OAK**	211.43 ± 0.635	212.93 ± 0.907	199.63 ± 1.527	0.105 ± 0.015	0.108 ± 0.003	0.094 ± 0.013
**F0 PG**	64.43 ± 1.730	64.35 ± 4.561	72.22 ± 1.191	0.060 ± 0.136	0.060 ± 0.014	0.177 ± 0.026
**F1 PG**	44.75 ± 1.523	43.85 ± 0.517	50.18 ± 0.893	0.056 ± 0.009	0.072 ± 0.011	0.070 ± 0.014
**F1 PG + BHT**	44.03 ± 1.129	40.54 ± 0.352	50.00 ± 1.008	0.038 ± 0.006	0.083 ± 0.005	0.093 ± 0.021
**F1 PG + ORE**	44.38 ± 0.779	46.47 ± 0.961	51.00 ± 1.260	0.062 ± 0.004	0.036 ± 0.019	0.083 ± 0.013
**F1 PG + EDTA**	54.58 ± 0.920	53.21 ± 1.472	60.16 ± 2.330	0.063 ± 0.013	0.063 ± 0.048	0.047 ± 0.012
**F1 PG + OAK**	43.55 ± 0.964	45.44 ± 0.645	52.35 ± 1.710	0.064 ± 0.011	0.081 ± 0.011	0.081 ± 0.014

*The results represent means ± standard deviations of three measurements.

Table S2. pH values and electrical conductivity of P80 and PG-based nanoemulsions during one month of storage at different temperatures.

	pH value			Electrical conductivity (µS/cm)		
**Formulation name**	5 °C	25 °C	40 °C	5 °C	25 °C	40 °C
**F0 P80**	5.32 ± 0.01	4.85 ± 0.01	3.39 ± 0.01	229.67 ± 1.155	190.53 ± 5.358	325.33 ± 0.578
**F1 P80**	5.70 ± 0.01	6.11 ± 0.06	3.72 ± 0.02	217.00 ± 3.464	155.50 ± 0.300	248.54 ± 0.777
**F1 P80 + BHT**	6.19 ± 0.04	6.16 ± 0.02	5.34 ± 0.01	199.67 ± 0.577	206.39 ± 2.310	179.23 ± 1.762
**F1 P80 + ORE**	6.31 ± 0.05	6.14 ± 0.06	4.84 ± 0.06	137.37 ± 0.115	169.87 ± 0.404	200.33 ± 0.577
**F1 P80 + EDTA**	6.37 ± 0.01	4.82 ± 0.03	4.77 ± 0.08	737.33 ± 1.155	749.33 ± 1.527	703.67 ± 0.345
**F1 P80 + OAK**	6.22 ± 0.02	6.21 ± 0.03	5.59 ± 0.03	263.83 ± 0.578	224.67 ± 0.577	270.33 ± 2.517
**F0 PG**	3.96 ± 0.03	3.98 ± 0.02	3.92 ± 0.01	246.67 ± 8.083	287.00 ± 3.464	300.33 ± 0.577
**F1 PG**	4.02 ± 0.03	4.07 ± 0.05	4.02 ± 0.02	85.83 ± 0.351	115.80 ± 1.114	89.73 ± 0.115
**F1 PG + BHT**	3.98 ± 0.01	3.98 ± 0.01	3.95 ± 0.06	81.42 ± 1.166	115.47 ± 1.569	118.07 ± 7.557
**F1 PG + ORE**	4.04 ± 0.05	4.05 ± 0.01	3.99 ± 0.08	84.70 ± 3.118	95.30 ± 2.995	103.30 ± 4.729
**F1 PG + EDTA**	4.80 ± 0.04	4.47 ± 0.06	4.41 ± 0.07	285.47 ± 1.790	356.00 ± 1.001	349.00 ± 1.125
**F1 PG + OAK**	4.60 ± 0.02	4.17 ± 0.03	3.99 ± 0.04	111.21 ± 1.607	108.10 ± 0.173	99.87 ± 1.270

*The results represent means ± standard deviations of three measurements.
